# Concurrent infiltration by CD8^+^ T cells and CD4^+^ T cells is a favourable prognostic factor in non-small-cell lung carcinoma

**DOI:** 10.1038/sj.bjc.6602934

**Published:** 2006-01-17

**Authors:** K Hiraoka, M Miyamoto, Y Cho, M Suzuoki, T Oshikiri, Y Nakakubo, T Itoh, T Ohbuchi, S Kondo, H Katoh

**Affiliations:** 1 Surgical Oncology, Cancer Medicine, Division of Cancer Medicine, Hokkaido University Graduate School of Medicine, Kita-15, Nishi-7, Kita-ku, Sapporo 060-8638, Japan; 2 Department of Medicine, University of California Los Angeles, MRL-1551, 675 Charles E Young Drive South, Los Angeles, CA 90095, USA; 3 Department of Surgical Pathology, Hokkaido University Hospital, Kita-15, Nishi-7, Kita-ku, Sapporo 060-8638, Japan;; 4 Department of Thoracic Surgery, Minami-Ichijo Hospital, Minami-1, Nishi-13, Chuo-ku, Sapporo 060-0061, Japan

**Keywords:** non-small-cell lung carcinoma, prognostic factor, CD8^+^ T cells, CD4^+^ T cells, immunohistochemistry

## Abstract

The purpose of this study was to clarify the relationship between the number of tumour-infiltrating T lymphocytes and the clinicopathological features and clinical outcome in patients with non-small-cell lung cancer (NSCLC). Tissue specimens from 109 patients who underwent surgical resection for NSCLC were immunohistochemically analysed for CD4 and CD8 expression. Patients were classified into two groups according to whether their tumours exhibited a ‘high’ or ‘low’ level of CD8^+^ or CD4^+^ lymphocyte infiltration. Although the level of infiltration by CD8^+^ T cells alone had no prognostic significance, the survival rate for patients with both ‘high’ CD8^+^ and ‘high’ CD4^+^ T-cell infiltration was significantly higher than that for the other groups (log-rank test, *P*=0.006). Multivariate analysis indicated that concomitant high CD8^+^ and high CD4^+^ T-cell infiltration was an independent favourable prognostic factor (*P*=0.0092). In conclusion, the presence of high levels of both CD8^+^ T cells and CD4^+^ T cells is a significant indicator of a better prognosis for patients with NSCLC, and cooperation between these cell populations may allow a significantly more potent antitumour response than either population alone.

Lung cancer is one of the most common malignancies in the world, and despite remarkable advances in diagnosis and treatment for the disease, the prognosis in most cases remains poor. Non-small-cell lung cancer (NSCLC) represents about 75–80% of all lung cancers, and its overall 5-year survival rate is less than 12–15% (Ihde, 1992; Non-small Cell Lung Cancer Collaborative Group, 1995; Jemal *et al*, 2005). These facts make clear the need for new parameters that will allow better prognostic evaluation for this malignancy and more reliable identification of patients likely to benefit from adjuvant therapy.

In a variety of human solid cancers, tumour-infiltrating T lymphocytes (TILs) are considered to play important roles in anticancer immunomechanisms of the tumour-bearing host. Among TILs, most CD8^+^ T cells are cytotoxic T lymphocytes that recognise particular tumour-associated antigens presented on MHC class I molecules at the cancer cell surface and possess the ability to destroy cancer cells directly. The favourable prognostic significance of the presence of tumour-infiltrating CD8^+^ T cells was previously reported in a variety of cancers, including colorectal cancer, oesophageal cancer, pancreatic cancer, bile duct cancer and gallbladder cancer (Naito *et al*, 1998; Nakano *et al*, 2001; Schumacher *et al*, 2001; Cho *et al*, 2003; Nakakubo *et al*, 2003; Oshikiri *et al*, 2003; Fukunaga *et al*, 2004). In NSCLC, however, the relationship between patient prognosis and the presence of TILs is still obscure. The infiltration of natural killer cells or macrophages into tumours was previously found to indicate a favourable prognosis (Takeo *et al*, 1986; Takanami *et al*, 2001), whereas the presence of tumour-infiltrating CD8^+^ T cells was not found to be a predictor of patient survival (Mori *et al*, 2000; Wakabayashi *et al*, 2003).

CD4^+^ T cells play a central role in orchestrating the immune response to cancer. Essentially, CD4^+^ T cells recognise peptides presented on MHC class II molecules expressed primarily on antigen-presenting cells. Although most tumour cells do not express MHC class II molecules, CD4^+^ T cells can effect an antitumour response in the absence of CD8^+^ T cells by secreting cytokines, such as interferon-*γ* (Mumberg *et al*, 1999; Qin and Blankenstein, 2000), or by activation and recruitment of effector cells such as macrophages and eosinophils (Greenberg, 1991; Hung *et al*, 1998). However, the main role of CD4^+^ T cells in the immune response to cancer is to prime CD8^+^ cells and maintain their proliferation.

The purpose of the present study was to evaluate the infiltration of CD4^+^ and CD8^+^ T cells by immunohistochemistry in order to clarify the individual or synergistic role of TILs in NSCLC.

## MATERIALS AND METHODS

### Patients and tissue specimens

Tissue specimens from 109 patients who underwent surgical resection for NSCLC between 1994 and 1996 in the Department of Thoracic Surgery at Minami-Ichijo Hospital were studied. These cases were histopathologically composed of 58 cases of adenocarcinoma, 40 of squamous cell carcinoma, seven of large cell carcinoma, three of adeno-squamous cell carcinoma and one of carcinosarcoma. Surgical resection was not performed in patients presenting with distant site metastases. None of these patients received either preoperative radiation or chemotherapy. Our series included 71 men and 38 women with a mean age of 63 years (range 39–80). Follow-up data for each of these patients was collected for at least 5 years, and 52 patients (48%) died during the follow-up period. All specimens were fixed in 10% formalin and embedded in paraffin wax. Unstained 4-*μ*m sections were then cut from paraffin blocks for immunohistochemical analysis. Histological classification of tumours was based on the World Health Organization criteria. All tumours were staged according to the pTNM pathological classification of the UICC (International Union Against Cancer) (Sobin and Wittekind, 1997).

### Immunohistochemical examination

For immunohistochemical analysis, formalin-fixed and paraffin-embedded sections were deparaffinised in xylene and rehydrated through a graded series from ethanol to water. Endogenous peroxidase activity was blocked by incubation in 3% hydrogen peroxide for 10 min. After washing in phosphate-buffered saline (PBS, pH 7.4) twice, the sections were incubated with 10% normal goat serum for 5 min and then incubated overnight at 4°C with the primary antibodies at the following dilutions: anti-CD8 mouse monoclonal antibody (C8/144B; DAKO, Glostrup, Denmark) 1 : 50, anti-CD4 mouse monoclonal antibody (1F6; Novocastra, Newcastle, UK) 1 : 50. For negative controls, we used 10% normal mouse serum in place of primary antibody. After three additional washes, a biotinylated goat anti-mouse antibody (Histofine SAB-PO kit; Nichirei Co., Tokyo, Japan) was applied at room temperature for 30 min. After the sections were washed in PBS three times, freshly prepared 3,3′-diaminobenzidine tetrahydrochloride (Histofine Simple stain DAB Solution; Nichirei Corporation, Tokyo, Japan) was used to visualise antibody binding and the sections were washed in distilled water. Sections were counterstained with haematoxylin and mounted in Permount.

### Evaluation and classification of CD8^+^ and CD4^+^ T cells

Tumour-infiltrating CD8^+^ T cells were classified into two groups by their location: CD8^+^ T cells within cancer stroma adjacent to cancer cell nests, or CD8^+^ T cells within the cancer cell nests themselves. For each group, we counted the number of CD8^+^ T cells with a magnification of × 200 (Olympus Optical Co., Ltd, Tokyo, Japan). The number of CD4^+^ T cells in cancer stroma were also counted and classified in the same way. Five areas containing the highest abundance of TILs were evaluated in each case. All counting was performed independently by three investigators of this study without knowledge of the patients' background or outcome, and immunoreactivity in each section was represented by the median scores.

### Statistical analysis

Correlations between the numbers of both CD8^+^ T cells and CD4^+^ T cells with the patients' clinicopathological variables were analysed by the *χ*^2^ test or Fisher's exact probability test. Correlation coefficient was described as *r*, and *r*>0.7 was defined as a strong correlation. Univariate analysis of the survival data was performed using survival curves that applied the Kaplan–Meier method with log-rank analysis. The influence of variables on survival was assessed using Cox multivariate regression analysis. The risk ratio and its 95% confidence interval were recorded for each marker. Probability values of less than 0.05 were considered statistically significant in all analyses. All statistical analysis was performed using StatView version 5.0 (SAS Institute Inc., Cary, NC, USA).

## RESULTS

### Immunohistochemistry and classification

**Figure 1:**
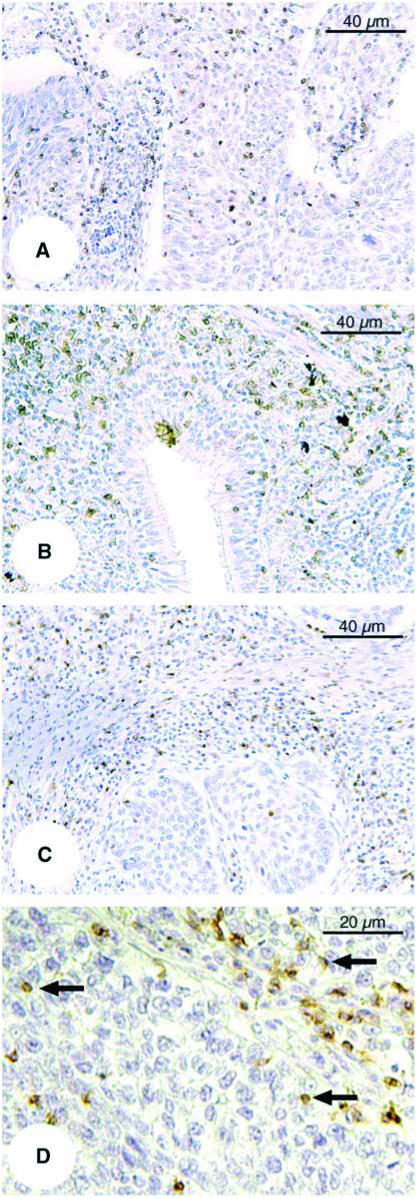
Representative immunohistochemical staining of NSCLC tumour sections. (**A**) Adenocarcinoma (anti-CD8 antibody; original magnification, × 200). (**B**) Adenocarcinoma (anti-CD4 antibody, × 200). (**C**) Squamous cell carcinoma (anti-CD8 antibody, × 200). (**D**) Squamous cell carcinoma (anti-CD8 antibody, × 400). Arrows indicate CD8-positive stained lymphocytes.

Tumour-infiltrating T lymphocytes were predominantly observed within the cancer stroma by immunohistochemical staining for CD8 and CD4 (Figure 1). The number of infiltrating CD8^+^ or CD4^+^ T cells in cancer stroma was overwhelmingly higher than that within cancer nests.

**Figure 2:**
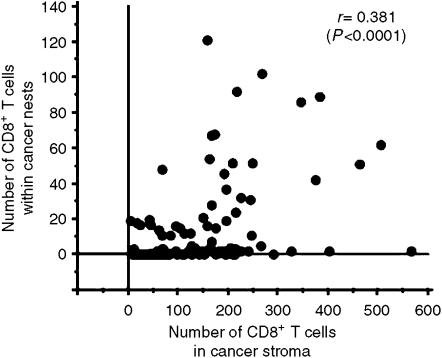
The correlation between the number of CD8^+^ T cells in cancer stroma and within cancer cell nests in patients with NSCLC.

Among 109 NSCLC specimens, the mean number of infiltrating CD8^+^ T cells in cancer stroma was 152±107 (median 148, range 6–566), that of CD8^+^ T cells within cancer nests was 14±25 (median 2, range 0–121) and that of CD4^+^ T cells in cancer stroma was 169±152 (median 134, range 0–626). The number of CD8^+^ T cells in the stroma and CD8^+^ T cells within cancer nests of individual cases are shown in Figure 2. There was no significant correlation (*r*=0.381; *P*<0.0001) between the number of CD8^+^ T cells in the stroma and the number of CD8^+^ T cells within cancer cell nests. We utilised the mean number of infiltrating cells as a cutoff point to divide all tumours into groups as having either ‘high’ or ‘low’ infiltration by CD8^+^ and CD4^+^ cells in stromal and in nest tissue. Fifty-three cases were classified as having ‘high’ (>151) levels of CD8^+^ T-cell infiltration in stromal tissue and the remainder as having ‘low’ (0–151) infiltration. Thirty-one cases were classified as having ‘high’ (>14) CD8^+^ T-cell infiltration within cancer nests and the remainder as having ‘low’ (0–14) infiltration. Forty-four cases were classified as having ‘high’ (>168) CD4^+^ T-cell infiltration within stroma and the remainder as having ‘low’ (0–168) infiltration.

### Relationships between CD8^+^ TILs and clinicopathological variables

The relationships between the levels of infiltration by CD8^+^ T cells in tumour stroma or nests and various clinicopathological features are summarised in Table 1. Significant correlations with the number of CD8^+^ T cells in cancer stroma were noted for lymph node metastasis and pTNM stage (*P*=0.019 and 0.033, respectively). However, no significant correlations were found between the number of CD8^+^ T cells in cancer stroma and age, gender, pT classification, histopathological grading and histological type. On the other hand, the number of CD8^+^ T cells within cancer cell nests was significantly correlated with gender (*P*=0.003), histopathological grading (*P*=0.047) and histological type (*P*<0.0001). We also found that poorly differentiated tumours or squamous cell carcinomas showed significantly higher numbers of CD8^+^ T-cell infiltration within cancer nests than did well-differentiated tumours or tumours of other histological types. There was a strong correlation between gender (males) and histological type (squamous cell carcinoma) in the patient cohort of this study (*P*<0.0001). No significant correlations were found between the level of CD4^+^ T-cell infiltration in cancer stroma and any of the examined clinicopathological variables (data not shown).

### Kaplan–Meier survival analysis

**Figure 3:**
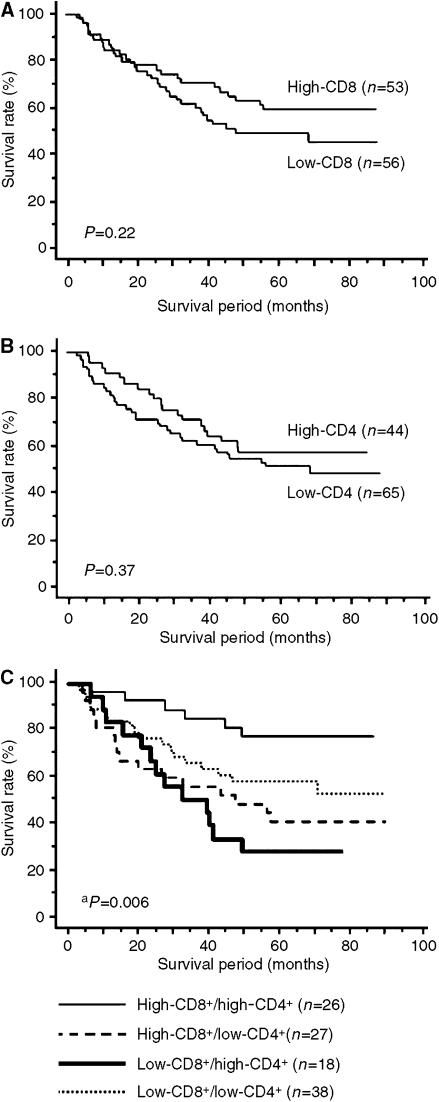
Kaplan–Meier analysis of overall survival according to (**A**) the level of infiltration by CD8^+^ T cells in cancer stroma, (**B**) the level of infiltration by CD4^+^ T cells in cancer stroma and (**C**) the simultaneous presence of high levels of infiltrating CD8^+^ T cells and CD4^+^ T cells in cancer stroma in patients with NSCLC. For details about the method of classification, see Materials and Methods. ^a^High-CD8^+^/high-CD4^+^ group (*n*=26) *vs* others (*n*=83).

Kaplan–Meier analysis revealed that the level of CD8^+^ T cells within cancer nests or in cancer stroma or CD4^+^ T cells in cancer stroma independently had no significant relationship to the survival of the patients. Survival curves constructed using the Kaplan–Meier method are shown in Figure 3. Among the 109 patients of our study, no significant difference in survival was observed between those with ‘high’ or ‘low’ levels of CD8^+^ T cells in cancer stroma (Figure 3A). Similarly, the level of stromal infiltration by CD4^+^ T cells demonstrated no prognostic significance (Figure 3B). To evaluate the possibility that a high level of both CD8^+^ and CD4^+^ T-cell infiltration in cancer stroma might correlate with favourable patient prognosis, the patients were classified into four groups: high-CD8^+^/high-CD4^+^ T cells (*n*=26), high-CD8^+^/low-CD4^+^ (*n*=27), low-CD8^+^/high-CD4^+^ (*n*=18) and low-CD8^+^/low-CD4^+^ T cells (*n*=38). The group that exhibited both highCD8^+^ and high CD4^+^ T-cell infiltration in cancer stroma demonstrated significantly higher survival rates than the rest of the patients (log-rank test, *P*=0.006; Figure 3C).

### Multivariate analysis of CD8^+^/CD4^+^ T cells and clinicopathological variables

Multivariate analysis of the patients as grouped in the same way was performed with other clinicopathological predictors for survival time using the Cox regression model (Table 2). The results indicated that high-CD8^+^/high-CD4^+^ T cells infiltration in cancer stroma was an independent favourable prognostic factor (risk ratio, 3.221; *P*=0.0092). Positive lymph node metastasis and pT classification (pT2–4) also had independent unfavourable prognostic values, with a risk ratio of 2.410 (*P*=0.0036) and 4.502 (*P*<0.0001), respectively. Although histological type had prognostic significance by univariate analysis, no significance was observed by multivariate analysis.

## DISCUSSION

In the immune response to cancer cells, tumour-infiltrating CD8^+^ T cells play an essential role, recognising tumour-associated antigens presented on MHC class I molecules expressed on the cancer cell surface and directly lysing cancer cells expressing the same antigens. In previous immunohistochemical studies in a variety of cancers, larger numbers of tumour-infiltrating CD8^+^ T cells usually signified a stronger immune reaction against the cancer and indicated a better prognosis. In colorectal carcinomas, the presence of large numbers of CD8^+^ T cells within cancer cell nests was a favourable independent prognostic factor (Naito *et al*, 1998), and a similar result was observed in oesophageal carcinoma (Schumacher *et al*, 2001). Furthermore, we previously demonstrated the prognostic significance of infiltrating CD8^+^ T cells in pancreatic cancer, gallbladder cancer, bile duct cancer and oesophageal squamous cell carcinoma (Cho *et al*, 2003; Nakakubo *et al*, 2003; Oshikiri *et al*, 2003; Fukunaga *et al*, 2004). However, in NSCLC, the role of TILs for the survival of patients is still controversial. Consistent with the previous results (Mori *et al*, 2000; Wakabayashi *et al*, 2003), the present study demonstrated that neither CD8^+^ T cells within cancer cell nests nor those in cancer stroma had a significant impact on patient survival. The reasons for this discrepancy were difficult to explain, because the antitumour effect of CD8^+^ T cells may be circumvented by various mechanisms in the tumour cells. Tumour cells may obtain the ability to evade immune surveillance by several strategies, including a lack of adequate T-cell costimulation (Melero *et al*, 1997; Thomas *et al*, 1999), downregulation of cell-surface MHC class I expression (Goodenow *et al*, 1985; Restifo *et al*, 1993; Imboden *et al*, 2001), dysfunction of Fas (CD95/APO1)-mediated apoptosis (Hahne *et al*, 1996; Strand *et al*, 1996) and secretion of immunosuppressive factors, such as transforming growth factor-*β* (Inge *et al*, 1992) or interleukin-10 (Fiorentino *et al*, 1991a, 1991b). Thus, the efficiency of the immune reaction against cancers can be evaded by a variety of mechanisms used by tumour cells, and these can vary, depending on the individual cancer. It will be worthwhile to investigate the effect of such immuno-evasive factors on the ability of CD8^+^ T cells to mount an effective response against tumour cells in NSCLC.

Despite the fact that we observed no prognostic significance of high CD4^+^ T-cell infiltration alone, we found a synergistic effect of simultaneous high CD4^+^ T-cell and CD8^+^ T-cell infiltration in cancer stroma as a favourable prognostic factor in NSCLC. In the presence of a relatively high level of CD4^+^ T-cell infiltration, patients with a sufficient number of tumour-infiltrating CD8^+^ T cells demonstrated a significantly better prognosis. We have previously demonstrated a similar synergistic effect between tumour-infiltrating CD8^+^ T cells and CD4^+^ T cells in oesophageal squamous cell carcinoma and pancreatic adenocarcinoma (Cho *et al*, 2003; Fukunaga *et al*, 2004). The present study is the first report demonstrating such an effect directly observed in resected human specimens. This result suggests that cooperation between infiltrating CD4^+^ T cells and CD8^+^ T cells in tumours might be important in the suppression of the progression of NSCLC. Previous studies have demonstrated that activation of CD4^+^ T cells is required for immunisation of CD8^+^ T cells against cancer. For activation and maintenance of tumour-infiltrating CD8^+^ T cells, CD4^+^ T cells play an important role by secreting cytokines such as interleukin-2, which is required for CD8^+^ T-cell growth and proliferation (Rosenberg *et al*, 1988, 1998; Yoshino *et al*, 1992; Cheng *et al*, 1998; Wang and Rosenberg, 1999; Rosenberg, 2001; Wang, 2001; Zeng, 2001). Reduction of CD4^+^ T lymphocytes by administration of anti-CD4 antibody allowed human lung cancer xenografts to form orthotopically in immuno-competent mice (Hunt *et al*, 2000). As CD4^+^ T cells are necessary for the full antitumour activity of CD8^+^ T cells, this may explain why a high level of CD8^+^ T-cell infiltration alone in this study did not correlate with a more favourable prognosis.

In the present study, we demonstrated that the level of CD8^+^ T-cell infiltration in cancer stroma correlated with pTNM staging and the presence of lymph node metastasis. Although multivariate analysis demonstrated that the presence of lymph node metastasis was a significant independent unfavourable prognostic factor, this result suggests that tumour-infiltrating CD8^+^ T cells may not only locally but also systemically suppress metastasis in lymph tracts.

Moreover, we found that the level of CD8^+^ T-cell infiltration within cancer cell nests had significant correlations with tumour dedifferentiation and histological subtype in NSCLC, consistent with previous reports (Mori *et al*, 2000; Wakabayashi *et al*, 2003). One possible reason for these results is a difference in immunogenicity of each subtype of cancer. In NSCLC, poorly differentiated cancers or squamous cell carcinomas may express larger amounts of tumour-associated antigens than other types of cancers, and hence, the number of CD8^+^ T cells within cancer cell nests might reflect only the peculiarities of the cancer cells in small locoregional areas. As the average number of CD8^+^ T cells within cancer cell nests was over 10-fold lower than that in cancer stroma in the present data, CD8^+^ T cells within cancer cell nests might not have a significant influence on the immune response against cancer due to the low level of antitumour immunity.

In conclusion, we found that infiltrating CD8^+^ T cells and CD4^+^ T cells in NSCLC may cooperate to suppress cancer progression and their presence together appears to be an independent favourable prognostic factor in this disease. The presence of both infiltrating CD8^+^ T cells and CD4^+^ T cells in resected specimens may be a useful index for adjuvant therapies for patients with poor prognosis.

## Figures and Tables

**Table 1:** Relationship between CD8^+^ T-cell infiltration and clinicopathological features in NSCLC

	**CD8^+^ T cells in cancer stroma**			**CD8^+^ T cells within cancer nests**		
**Variables**	**Low (*n*=56)**	**High (*n*=53)**	***P***-**value^a^**	**Low (*n*=78)**	**High (*n*=31)**	***P***-**value^a^**
*Age (years)*						
<64	31	23	0.141	41	13	0.317
⩾64	25	30		37	18	

*Gender*						
Male	36	35	0.848	44	27	0.003
Female	20	18		34	4	

*pT classification*						
pT1	27	20	0.270	37	10	0.149
pT2–4	29	33		41	21	

*Lymph node metastasis*						
Negative	34	43	0.019	56	21	0.675
Positive	22	10		22	10	

*pTNM stage*						
I	29	38	0.033	51	16	0.183
II–III	27	15		27	15	

*Tumour grade*						
Poor	4	9	0.113	6	7	0.047
Other	52	44		72	24	

*Histological type*						
Squamous cell carcinoma	21	19	0.858	19	21	<0.0001
Other	35	34		59	11	

a *P*-value was calculated by *χ*^2^ test or Fisher's exact test.

NSCLC=non-small-cell lung cancer.

**Table 2:** Univariate and multivariate analyses of T-cell infiltration status and clinicopathological features in 109 patients with NSCLC

	**Kaplan–Meier (log rank)**	**Multivariate analysis**	
**Variables**	***P***-**value**	**Hazard ratio (95% CI)**	***P***-**value**
pT classification (2–4 *vs* 1)	<0.0001	4.502 (2.17–9.34)	<0.0001
Lymph node metastasis (positive *vs* negative)	<0.0001	2.410 (1.33–4.35)	0.0036
Histological type (sqamous *vs* other)	0.0001	1.726 (0.95–3.13)	0.729
CD8^+^/CD4^+^ T-cell infiltration (other *vs* high/high)	0.006	3.221 (1.34–7.77)	0.0092

NSCLC=non-small-cell lung cancer; CI=confidence interval.
